# Influence of Different Beam Oscillation Patterns in Electron Beam Welding of Niobium Sheets with Different Thickness

**DOI:** 10.3390/ma15113778

**Published:** 2022-05-25

**Authors:** Jia Tao, Jiefeng Wu, Zhihong Liu, Jianguo Ma, Zhenfei Liu, Wuqingliang Peng

**Affiliations:** 1 Institute of Plasma Physics, HFIPS, Chinese Academy of Sciences, Hefei 230031, China; whyztj123456@163.com (J.T.); jfw@ipp.ac.cn (J.W.); zhliu@ipp.ac.cn (Z.L.); qingliang.pengwu@ipp.ac.cn (W.P.); 2 Science Island Branch, University of Science and Technology of China, Hefei 230026, China; 3 Anhui Province Key Laboratory of Special Welding Technology, Huainan 232000, China; zhenfei.liu@ipp.ac.cn

**Keywords:** iris weld, niobium sheets, electron beam welding, oscillating patterns, EBSD, mechanical properties, low temperature

## Abstract

The electron beam welding of the tubes and the half-cells for our 1.3 GHz single-cell superconducting radiofrequency (SRF) cavities is complex due to the different thicknesses of the tubes and the half-cells in the iris region. However, the mechanical properties and microstructure of the iris welds in niobium SRF cavities have barely been explored in previous studies. For high-quality iris welds, welding experiments of niobium sheets of 2 mm and 2.8 mm were carried out under different oscillating conditions. The results show that welding with no oscillation or sinusoidal oscillation may not be applied in actual welding owing to the large misalignment of the bottom surface. The weld grains were not significantly refined through beam oscillation. The joints with infinity oscillation had a higher elongation than circular oscillation, which exhibited a brittle fracture in the tensile tests at 77 K. Nevertheless, the texture of the weld with infinity oscillation implies poor formability, so the feasibility of infinity oscillation in actual welding needs verification in future study.

## 1. Introduction

Superconducting radiofrequency (SRF) cavities, fabricated by niobium with high purity, have been the core components of particle accelerators for many years. Vacuum electron beam welding (EBW) has been applied in the fabrication of the SRF cavities owing to the limit of the gas content in the fusion zone (FZ) [1]. The welding of the tube and the half-cell, one of the fabrication steps of the cavities [2], is performed in the iris region, the small diameter of the half-cell [3]. The radius of the iris has a remarkable impact on the ratio of the surface peak electric field to the accelerating gradient (E_peak/_E_acc_) [4,5], which is closely related to the loss of the SRF cavity [6]. In addition, the welding distortion in the iris region probably makes the frequency of the cavity unable to meet the requirement [7]. Hence, the welding distortion in this region should be restricted to a certain extent. Furthermore, the electric field in the iris region is the highest [8], and thus it was proposed that the iris weld should be made partially from the outside and partially from the inside [2,8,9] at a certain angle with the axis of rotation [10] to guarantee a smooth inner surface in the region. An inferior surface finish in the iris region is likely to disturb the internal electric field. Most important of all, for a welded structure in service at low temperature, the mechanical properties, especially strength and ductility [11], are essential in evaluating the welding quality of the joints. What is more, grain structure and crystallographic texture are associated with mechanical properties [12,13,14]. According to the previous study [15,16,17], the grain structure and texture of the fusion zone and the heat-affected zone in the niobium joints after EBW may have a difference with the base metal due to different thermal processes in these regions, so the microstructure for niobium joints needs further investigation. However, in the previous research [18,19,20,21], the effect of weld defects and impurities on the superconducting properties of niobium welds has attracted more attention. The mechanical properties and microstructure of the iris welds have barely been explored so far. For our 1.3 GHz, single-cell SRF cavities applied in synchrotron radiation source devices, the thickness of the beam tubes and the irises of the half-cells are not the same, deviating from the structure of the reported cavities. The different thicknesses of these two parts may bring about significant welding stress and distortion [22] in the iris region. The mechanical properties of this iris weld are more difficult to predict, making this welding more challenging.

Beam oscillation has been commonly applied in the high-energy beam welding of various alloys to improve weld formation, solidified structure, and mechanical properties. In recent years, many reports have focused on the effect of beam oscillation on the laser beam welding process. Guoqing Chen et al. [23] found that the sizes of columnar crystals in the weld of thick-plate 2A12 aluminum alloy with circular beam oscillation were drastically reduced, and the strength of the joints was higher compared with that without beam oscillation, but Babu et al. [24,25] found that the strength was lowered when beam oscillation was adopted in the welding of Ti-6Al-4V. The ductility of this joint was enhanced when sinusoidal, square, and triangular oscillation were applied, whereas the structure and properties of these joints had no significant difference. Wenchao Ke [26] and Zhimin Wang et al. [27] suggested that the laser welding of 5A06 aluminum alloy with infinity oscillation reduced the porosities and avoided undercuts. Chen Zhang [28] and Zhenguo Jiang et al. [29] proposed that beam scanning tends to flatten the temperature gradient in weld pools, thereby assisting the growth of equiaxed grains. For the EBW of pure niobium sheets with unequal thickness, beam oscillation may also improve the weld quality according to the previous research, whereas few relevant experiments were carried out to investigate the effect of beam oscillation on niobium welds.

In the present work, the impact of different oscillating patterns on the welding process of niobium sheets with unequal thickness has been systematically investigated by EBSD analysis, tensile tests at low temperature, and microhardness tests. This research could provide a theoretical basis for the subsequent welding of the iris region in our single-cell SRF cavities.

## 2. Materials and Methods

**Figure 1:**
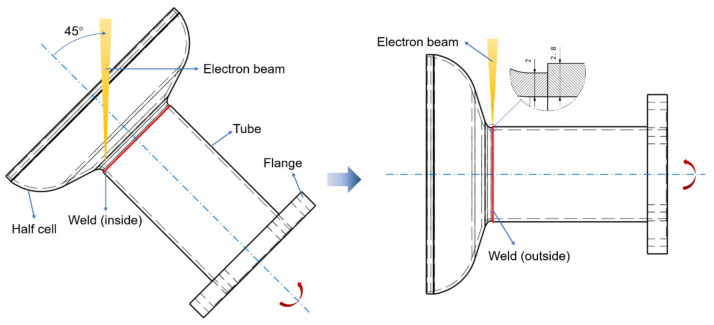
Sketch of EBW for iris weld.

**Figure 2:**
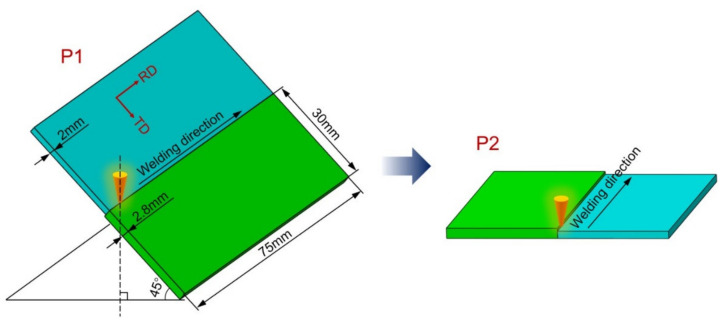
Schematic representation of butt welding on both sides.

**Figure 3:**
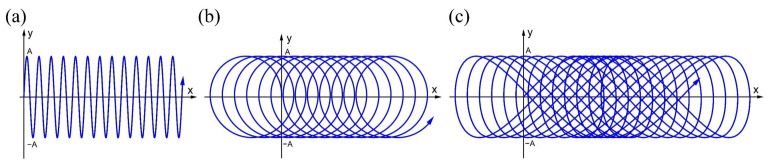
Different scanning paths for the welding tests: (**a**) sinusoidal path; (**b**) circular path; (**c**) infinity path.

The actual welding of the iris region is performed from the inside first in order to ensure the smoothness of the internal surface, then made from the outside with a partial penetration, as shown in Figure 1. Four pieces of rolled niobium sheets with a size of 75 × 30 × 2.8 mm^3^ and the others with a size of 75 × 30 × 2 mm^3^ were selected for EBW. The purity of these niobium sheets was 99.99%. Two pieces of the sheets with the thickness of 2 mm and 2.8 mm were butt welded at 45 degrees from the horizontal before the flat butt welding on the other side to simulate the inside and outside welding of the iris region, and the welding directions were identical at both sides, as depicted in Figure 2. The inclined position welding and the flat welding are marked as P1 and P2, respectively. It is worth noting that the two sheets’ top surfaces were in the same plane when P1 was performed. The welding experiment was carried out using a type ZD150-60C CV66M vacuum electron beam welding machine produced by Sea-Sun-Tech, Trappenkamp, Germany. The vacuum pressure was 9 × 10^−6^ mbar because the residual resistivity ratio (RRR) degradation was less than 10% when the pressure in the vacuum chamber was lower than 5 × 10^−5^ mbar [30]. For stage P1, a circular beam oscillation with a lesser beam current was adopted to reduce the weld bottom reinforcement, and this process is similar to tack welding [19]. In the practical welding of SRF cavities, it is significant to smooth the inner surface, where irregularity and defects can initiate a thermal breakdown [18]. Different beam oscillating patterns were applied in stage P2, including sinusoidal, circular, and infinity (∞), as depicted in Figure 3. The welding path equations of different patterns can be given as follows:

For linear path (no oscillation):

(1) $$x\left(t\right)=x_{0}+Vt,\;\;\;\;y\left(t\right)=y_{0}$$

For sinusoidal path:

(2) $$x\left(t\right)=x_{0}+Vt,\;\;\;\;y\left(t\right)=y_{0}+Asin\left(2\pi ft\right)$$

For circular path:

(3) $$x\left(t\right)=x_{0}+Vt+Acos\left(2\pi ft\right),\;\;\;\;y\left(t\right)=y_{0}+Asin\left(2\pi ft\right)$$

For infinity path:

(4) $$x\left(t\right)=x_{0}+Vt+2Asin\left(2\pi ft\right),\;\;\;\;y\left(t\right)=y_{0}+Asin\left(4\pi ft\right)$$

where *x*_0_ and *y*_0_ represent the initial position; *V* is the welding speed; and *A* and *f* are the oscillation amplitude and frequency, respectively. The welding parameters of these two stages referred to some previous successful welding experiments for niobium sheets [10], as shown in Table 1. Non-oscillation welding with the same parameters was taken as a comparison. It should be noted that the electron beam was focused above the sheets to reduce the spatter during welding.

**Figure 4:**
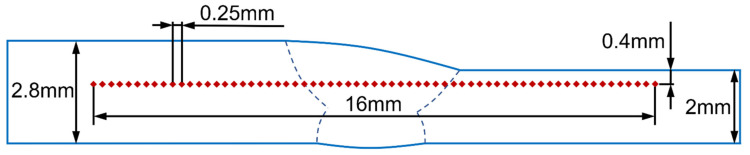
Schematic diagram of Vickers microhardness test.

**Figure 5:**
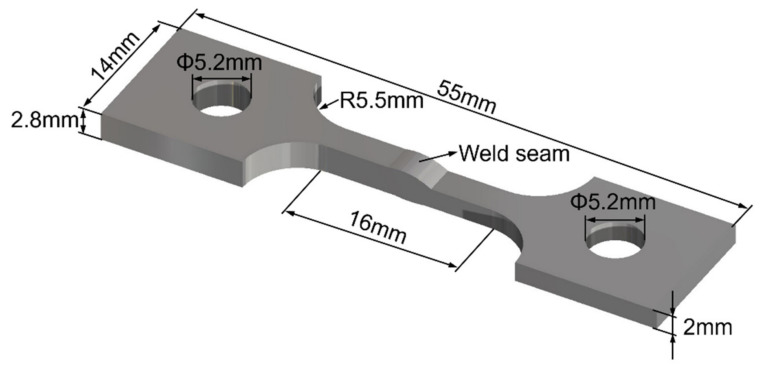
Schematic diagram of tensile test samples.

The welded samples and tensile test samples were sectioned by electro-discharge machining (EDM). The welded samples were first ground with SiC abrasive paper with grit sizes varying from 600 to 4000, then polished with 3 μm diamond suspension and 0.03 μm silica suspension. The morphology of the weld sections was observed after being etched with 8 mL HF, 6 mL H_2_SO_4_, 4 mL HNO_3_, and 20 mL distilled water. Subsequently, these samples were re-polished to prepare for electron back-scattered diffraction (EBSD) analysis. The EBSD was performed using a ZEISS Sigma 300 SEM. The microhardness of different joints was measured through a DHV-1000Z Vickers microhardness tester, with a loading load of 1.96 N and a hold time of 15 s. Since only the welding parameters of the top welds have changed, the straight-line region measured in this test is near the top surface, as shown in Figure 4. The tensile tests were carried out on a MTS-SANS CMT5000 machine at 77 K with a strain rate of 1 × 10^−3^ s^−1^ [31] due to the low operating temperature of SRF cavities. Three tensile specimens were prepared for each oscillating condition. The tensile samples were cut directly from the unequal thickness joints to evaluate the serviceability of these welded joints, as shown in Figure 5. The tensile fracture morphology was also studied using SEM after the tensile tests.

## 3. Results and Discussion

### 3.1. Formation Analysis

**Figure 6:**
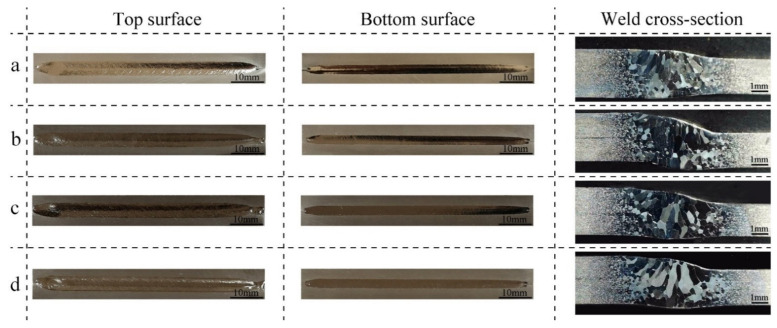
Surface and section morphology of the welded joints: (**a**) morphology of NEBW joint; (**b**) morphology of SEBW joint; (**c**) morphology of CEBW joint; (**d**) morphology of IEBW joint.

The top surface (welding surface in stage P2), the bottom surface (welding surface in stage P1), and the cross-sectional morphology of the welded joints with different scanning processes are shown in Figure 6. For simplicity, the EBW processes with no beam oscillation, sinusoidal, circular, and infinity oscillation were called NEBW, SEBW, CEBW, and IEBW, respectively. For all the joints, the surface was well-formed. No spatter, concave, or undercut defects were detected at the top and bottom surfaces of welded joints. This is probably because molten niobium has a large surface tension coefficient [32]. During EBW, the surface tension is balanced with recoil pressure [33], and thus larger surface tension tends to close the keyhole and make the flow in the molten pool less violent.

From the etched morphology of the weld cross-sections, it can be seen that all the joints were fully penetrated, and porosities are not present. The details of these sections are depicted in Table 2. As illustrated in Figure 6 and Table 2, the top of the weld and the HAZ of the joints with beam oscillation are slightly wider than those without oscillation due to the energy distribution in the joints with beam oscillation varying from directly welded joints. The widths of the weld top for the joints with oscillation are larger than those without oscillation, as most of the energy is focused on the edge of the molten pool instead of the weld center [34]. The similar weld widths of all the joints are primarily attributed to the small scanning amplitude and the same heat input [25]. The largest width of the CEBW joint is probably related to the distinctive flow behavior and lower cooling rate in the weld pool [35].

The width of the HAZ on the side with a thickness of 2 mm is larger than that with a thickness of 2.8 mm in all the joints because the cooling rate increases with the increase in sheet thickness without convection. The widths of the welds on the bottom surface are nearly the same owing to the identical welding parameters in stage P1. As the energy is not uniformly distributed on both sides of the joint with unequal thickness, misalignment occurs in the joints under nonuniform heat stress. The misalignment of the bottom surface in the NEBW and SEBW joints is more severe than that in the CEBW and IEBW joints for the different stress distribution caused by different scanning patterns [36]. Severe welding misalignment in the iris region greatly influences the frequency of the cavity, so welding with circular or infinity oscillation appears to be preferable for the iris weld to avoid large welding distortion. The weld bottom reinforcement of the SEBW joint is the largest among all the joints. Significant weld bottom reinforcement in the actual SRF cavity might disturb the internal magnetic and electric fields [8], harming the performance of the cavity.

### 3.2. Microstructure Analysis

**Figure 7:**
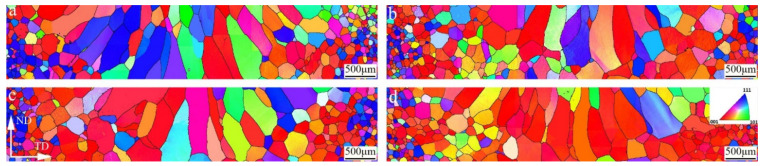
Inverse pole figure (IPF) coloring orientation diagram of welds and HAZ with different oscillation patterns: (**a**) grain structure of NEBW joint; (**b**) grain structure of SEBW joint; (**c**) grain structure of CEBW joint; (**d**) grain structure of IEBW joint.

**Figure 8:**
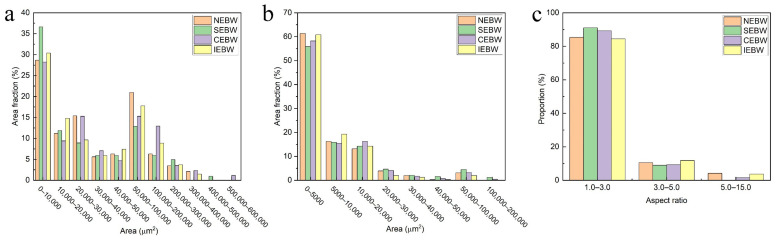
Comparison of grain size and aspect ratio: (**a**) grain size of FZ; (**b**) grain size of HAZ; (**c**) aspect ratio of FZ.

The solidification microstructure of the weld and the grain growth in HAZ are determined by different energy distributions of varying scanning patterns in the P2 stage. Figure 7 illustrates the grain structures of different joints. The columnar grains in the welds grew along the direction of heat dissipation. The grain size in the HAZ decreases gradually from the fusion line to the base metal, and the grains in the FZ grow epitaxially from the substrate. Figure 8 shows a comparison of the FZ and HAZ grain sizes with different oscillating patterns. Figure 8a suggests that the grains in the FZ were not obviously refined by beam oscillation. Some extremely large columnar grains with an area of over 400,000 μm^2^ were found in the SEBW and CEBW joints. Once beam oscillation is applied during EBW, the molten pool is enlarged, and the weld center is maintained at a higher temperature for a longer time, decreasing the solidifying and cooling rate [37]. Many nucleation sites are melted due to the prolonged holding time at a higher temperature. The remaining sites have sufficient time to grow into large grains. The SEBW and CEBW joints have larger welding widths, implying a longer holding time at higher temperatures, so some extremely large grains occurred in the FZ. As shown in Figure 8b, the grain coarsening of the HAZ in the SEBW and CEBW joints is more serious, which is related to the lower cooling rate in the HAZ. Figure 8c gives the aspect ratio of the weld grains. In general, the aspect ratio from large to small means converting the oriented columnar crystal structure into an equiaxed crystal structure, which is determined by a thermal gradient to solidification rate (G/R) [38]. When the temperature field reaches a quasi-steady state in the welding process, the solidification rate equals the welding speed [29], and thus the grain is only affected by the temperature gradient. A relatively moderate aspect ratio of weld grains in the SEBW and CEBW joints implies more flat temperature gradients in the weld pools of these two joints. In addition, the statistical data of the NEBW and IEBW joints suggests that the temperature gradient of the infinity scanning pool bears a closer resemblance to that of a no-scanning pool. However, the flow behaviors in the NEBW and IEBW are quite different, so the weld geometries of these two joints are inequable.

**Figure 9:**
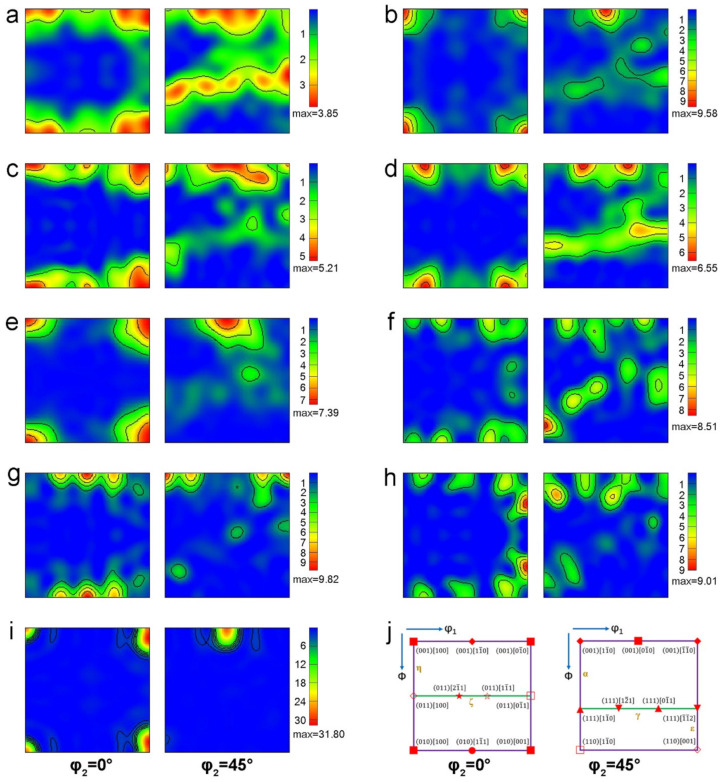
φ_2_ = 0° and 45° section ODFs of HAZ and FZ of the welded joints with different oscillating patterns: (**a**) base metal; (**b**) HAZ of NEBW joint; (**c**) HAZ of SEBW joint; (**d**) HAZ of CEBW joint; (**e**) HAZ of IEBW joint; (**f**) FZ of NEBW joint; (**g**) FZ of SEBW joint; (**h**) FZ of CEBW joint; (**i**) FZ of IEBW joint; (**j**) illustration of common texture components in bcc metals.

The evolution of the microstructure was obtained from orientation distribution function (ODF) maps. Figure 9 shows φ_2_ = 0° and 45° ODF sections of welded joints with different oscillating patterns. The initial texture of the base metal is characterized by {001}<100>, {111}<110>, and {111}<112> textures. The texture types of the HAZ are generally close to that of the base metal, but the texture intensity of the HAZ is higher than that of the base metal. The FZ exhibit a more complex and stronger texture. When no oscillating is applied during welding, the texture components in the weld contain {111}<112>, {155}<110>, and some {001}<120>. For the FZ of the SEBW joint, the main orientation is {001}<110>. A {001}<120> texture is also present in this weld. For the CEBW joint, the intensity of the {001}<140> texture was curtailed from the HAZ to the FZ, and the main orientation in the weld is {013}<031>. The results of the texture evolution are consistent with that of Kumar [39]. Due to the higher cooling rate of solidification for the FZ and the HAZ, the preferred orientation in these regions is stronger [16].

For the FZ of the IEBW joint, it has to be taken with caution that the intensity of the {001}<100> texture is much higher than that in the base metal and the HAZ. For the {001}<100>, {001}<110>, and {001}<120> textures, the plastic strain ratio (R-value) is not high compared with other textures according to the previous research [17,40]. In addition, the R values in the 0° and 90° directions differ greatly from that in the 45° direction for {001}<100> and {001}<110> textures, which is unfavorable for preventing unstable deformation in the welds of SEBW and IEBW joints. Additionally, the welds with {001}<100> and {001}<110> textures are prone to crack when the load in the thickness direction is strong.

### 3.3. Microhardness Profiles

**Figure 10:**
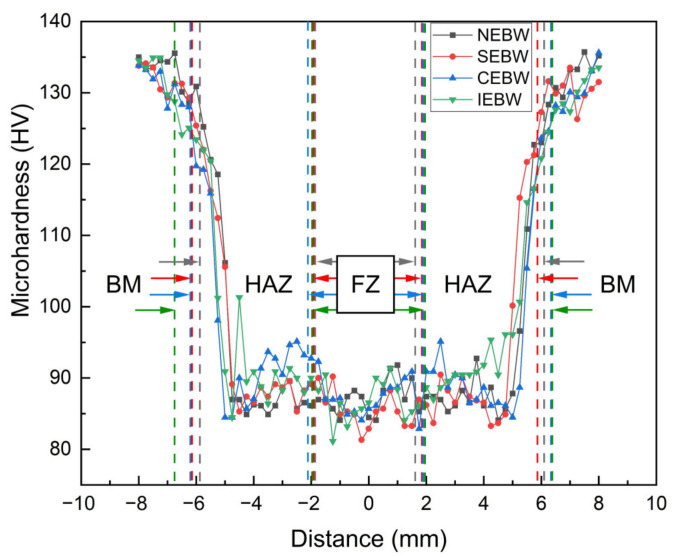
Microhardness profiles across the welded specimens from BM to FZ through HAZ.

The microhardness profiles for different oscillating patterns are plotted in Figure 10. Unlike welding power [17], oscillating pattern has little effect on the hardness distribution of the welded joints for niobium sheets due to the same heat input and no phase transition for all the joints [25]. The average microhardness values for the FZ of NEBW, SEBW, CEBW, and IEBW joints are 87.4, 85.6, 87.6, and 86.7, respectively. As indicated in Figure 8a, the average grain size for the FZ of the SEBW is large, so it has the lowest microhardness according to the Hall–Petch relationship [41]. The FZ of the CEBW is the hardest. This is probably because some grains with specific textures, such as {013}<031>, are harder in the rolling direction. The hardness distribution of the region close to the weld in the HAZ is almost the same as that of the FZ. At a certain distance from the fusion boundary, the hardness increases drastically with the decrease in the distance from the base metal. This observation coincides with the increasing grain size from the base metal to the FZ for all the joints.

### 3.4. Tensile Properties at 77 K

**Figure 11:**
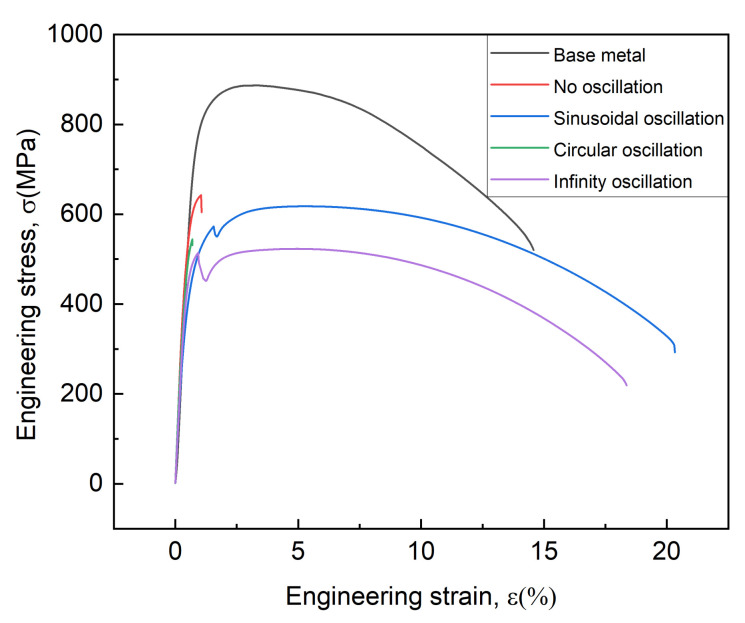
Engineering stress–strain curves of joints with different oscillating patterns during tensile testing at 77 K.

Figure 11 shows the tensile curves of the niobium base metal and welded joints at 77 K. The samples of welded joints were all fractured in the HAZ at the side with a thickness of 2 mm. The tensile curve for the base metal is relatively smooth, but sharp serrations occurred in the curves of the SEBW and IEBW samples. It may be associated with deformation twinning during tension [42]. The calculated tensile properties are shown in Table 3. All the welded joints showed lower tensile strength than the base metal. This result is consistent with that of Wu [43] and mainly attributed to the fine grains in the base metal referring to the Hall–Petch relationship [41] and no phase transition in the weld. The tensile and yield strength of the joints with no oscillation is the highest among all the joints. This result is in accordance with the grain size statistics in Figure 8. Almost all the welded joints with beam oscillation possess enhanced plasticity, except for the CEBW joint.

**Figure 12:**
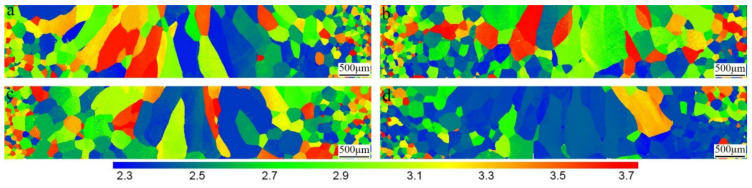
Taylor factor distribution of welded joints with different oscillation patterns: (**a**) NEBW joint; (**b**) SEBW joint; (**c**) CEBW joint; (**d**) IEBW joint.

**Figure 13:**
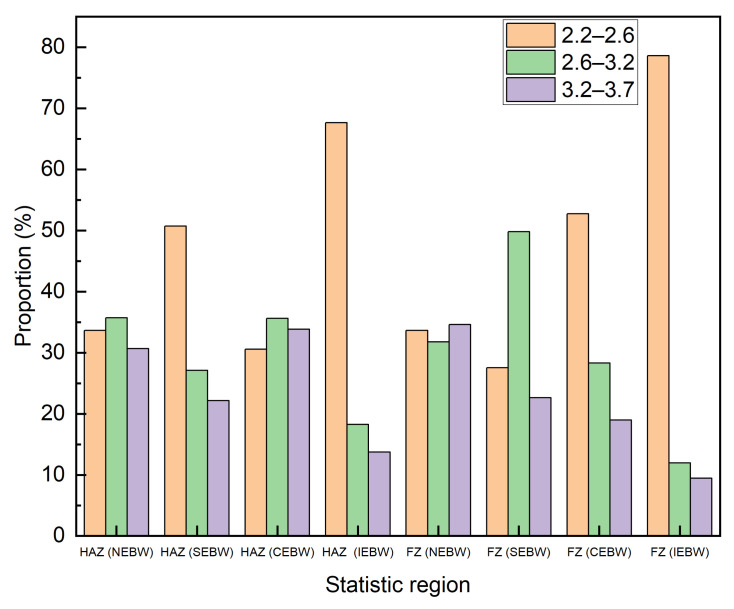
Fractions of grains with Taylor factor in HAZ and FZ of different joints.

The Taylor factor distribution obtained by the EBSD is also studied to evaluate the resistant ability to the plastic deformation of crystals [44]. Figure 12 shows the Taylor factor distribution maps for loading direction in the tensile tests. Three levels of the Taylor factor were defined according to the colors of the legend in Figure 12. The first level (2.2–2.6) contains grains that slip readily in the loading direction. The second level (2.6–3.2) consists of grains which have to deform in appropriate slip systems [45]. The third level (3.2–3.7) is marked from yellow to red, indicating that it is difficult to activate proper slip systems under load. It can be seen from Figure 12 and Figure 13 that the proportion of grains with the third level in the HAZ of the NEBW and CEBW joints is larger than that of the SEBW and IEBW joints, whereas it is the opposite for the proportion of grains with the first level in the HAZ. It can be deduced that the plastic deformation for the HAZ in the NEBW and CEBW joints is more difficult. The IEBW joint exhibits optimum plasticity. It is consistent with the highest Taylor factor in the HAZ and FZ of this joint. The distribution of Taylor factors is in accordance with the deformation behavior during tensile tests. The distribution of Taylor factors for different joints is associated with the grain growth process, which is affected by the different thermal processes of beam oscillation.

**Figure 14:**
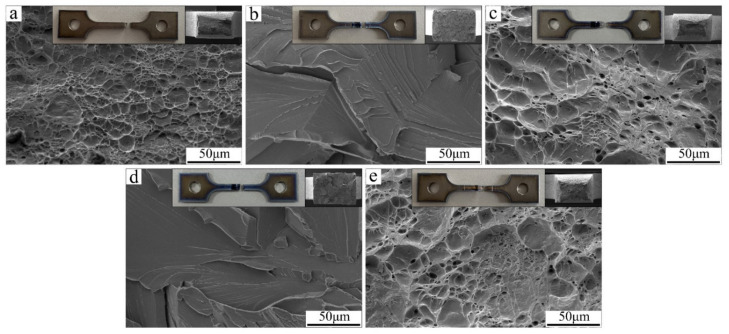
SEM fractography of tensile samples: (**a**) fracture surface of base metal; (**b**) fracture surface of NEBW sample; (**c**) fracture surface of SEBW sample; (**d**) fracture surface of CEBW sample; (**e**) fracture surface of IEBW sample.

Figure 14 shows the fracture surface morphology of tensile samples. The fracture surface of the SEBW joint, IEBW joint, and base metal is an oblique plane with many dimples. Many great dimples present tearing corrugates. The dimples in the fracture surface of the SEBW joint and IEBW joint are deeper than those of the base metal, indicating better plasticity of the SEBW and IEBW joints. For the NEBW and CEBW samples, the cleavage step and cleavage river patterns are obvious in the fracture surface. The area of sections was not reduced significantly, indicating a typical brittle fracture. Moreover, different residual stress distributions [46] after welding for these oscillating patterns may also influence the fracture types of the joints. This effect will be studied through simulation and measurement in the following research.

From the tensile properties and aforementioned analysis of weld formation and microstructure, it seems that the infinity oscillating pattern is the best choice for stage P2. However, the intense {001}<100> texture in the IEBW weld may bring about unstable deformation when this weld is loaded in multiple directions. What is more, the infinity oscillating process is less stable than the circular oscillating process according to the practical experience, especially for girth welding joints. Therefore, whether the infinity oscillation is exactly suitable for this welding has to be verified in the following girth welding experiments.

## 4. Conclusions

(1) Due to the large misalignment of the bottom surface, the joints welded with no oscillation or sinusoidal oscillation are probably not the candidate welding parameters for the electron beam welding of niobium sheets of 2 mm and 2.8 mm.
(2) The application of oscillation did not significantly refine the weld grains. The grains in the FZ and HAZ of joints are quite large when oscillating patterns are sinusoidal or circular, but the aspect ratio of grains in the fusion zone is relatively lower for these patterns.
(3) Intense {001}<100> and {001}<110> textures occurred in the weld when sinusoidal or infinity oscillation was applied, indicating poor formability of the fusion zone.
(4) Although the microhardness of different joints has no significant discrepancy, the joints welded with sinusoidal or infinity oscillation have superior plasticity at 77 K. The brittle fracture was determined for the joint welded with no oscillation or circular oscillation.
(5) The applicability of infinity oscillation to girth welding still requires verification.

## Figures and Tables

**Table 1:** EBW parameters at different stages.

Stage	Voltage U_a_/kv	Beam Current I_b_/mA	Focusing Current I_f_/mA	Velocity V/mm·s^−1^	Working Distance d/mm	Oscillation Frequency f/Hz	Oscillation Amplitude A/mm
P1	70	15	1545	6	300	300	1.1
P2	70	38	1500	6	300	300	1.3

**Table 2:** Weld geometries with different oscillating patterns.

Oscillating Pattern	Welding Width on Top Surface d_U_/mm	Welding Width on Bottom Surface d_L_/mm	Misalignment d_A_/mm	Weld Bottom Reinforcement h_L_/mm
No oscillation	4.45	3.01	0.34	0.11
Sinusoidal oscillation	4.64	2.99	0.44	0.27
Circular oscillation	4.73	2.97	0.03	0.18
Infinity oscillation	4.54	2.98	0.03	0.19

**Table 3:** Tensile properties of the base metal and welded joints with different oscillating patterns at 77 K.

Sample	Tensile Strength R_m_ (MPa)	Yield Strength (MPa)	Elongation δ (%)	Fracture Type
Base metal	886.5 ± 9.8	849.2 ± 11.5 (R_p0.2_)	9.5 ± 1.1	Ductile fracture
No oscillation	633.8 ± 16.2	582.3 ± 20.4 (R_p0.2_)	1.0 ± 0.2	Brittle fracture
Sinusoidal oscillation	609.2 ± 19.8	575.6 ± 15.5 (Upper yield point) 548.2 ± 19.3 (Lower yield point)	22.1 ± 2.6	Ductile fracture
Circular oscillation	549.33 ± 21.2	516.9 ± 10.6 (R_p0.2_)	0.7 ± 0.1	Brittle fracture
Infinity oscillation	517.2 ± 8.5	512.4 ± 13.8 (Upper yield point) 448.9 ± 8.7 (Lower yield point)	18.9 ± 1.8	Ductile fracture

## Data Availability

The data presented in this study are available on request from the corresponding author.
